# Exosomes as natural vectors for therapeutic delivery of bioactive compounds in skin diseases

**DOI:** 10.3389/fphar.2025.1485769

**Published:** 2025-04-28

**Authors:** Somayeh Keshtkar, Zahra Asvar, Haniyeh Najafi, Mozhdeh Heidari, Maryam Kaviani, Fatemeh Sabet Sarvestani, Ali Mohammad Tamaddon, Maryam Sadat Sadati, Nasrin Hamidizadeh, Negar Azarpira

**Affiliations:** ^1^ Molecular Dermatology Research Center, Shiraz University of Medical Sciences, Shiraz, Iran; ^2^ Nanotechnology School of Advanced Medical Sciences and Technologies, Shiraz University of Medical Sciences, Shiraz, Iran; ^3^ Department of Pharmaceutical Nanotechnology, Center for Nanotechnology in Drug Delivery, Shiraz University of Medical Sciences, Shiraz, Iran; ^4^ Transplant Research Center, Shiraz University of Medical Sciences, Shiraz, Iran

**Keywords:** exosomes, delivery, skin diseases, drug, genetic agent, protein

## Abstract

Skin diseases are a broad category of diseases and each has complex conditions, which makes it challenging for dermatologists to provide targeted treatment. Exosomes are natural vesicles secreted by cells and play a key role in cell communication. Due to their unique characteristics, including inherent stability, minimal immunogenicity, high biocompatibility, and exceptional ability to penetrate cells, exosomes are being explored as potential delivery vehicles for therapeutics across various diseases including skin problems. Utilizing exosomes for drug delivery in skin diseases can improve treatment outcomes and reduce the side effects of traditional drug delivery methods. Indeed, exosomes can be engineered or utilized as an innovative approach to deliver therapeutic agents such as small molecule drugs, genes, or proteins specifically to affected skin cells. In addition to targeting specific skin cells or tissues, these engineered exosome-based nanocarriers can reduce off-target effects and improve drug efficacy. Hence, this article highlights the transformative potential of this technology in revolutionizing drug delivery in dermatology and improving patient outcomes.

## Graphical Abstract

**Figure:**
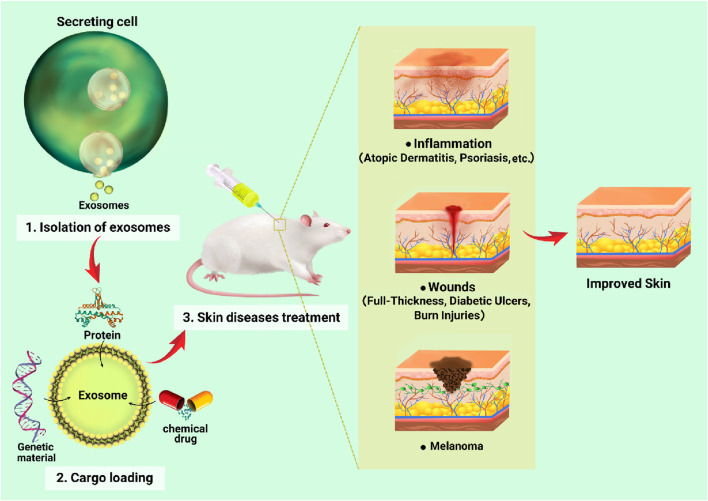
Summary of the application of exosomes for delivery of genetic agents, small molecule drugs, proteins in skin diseases.

## 1 Introduction

Skin diseases can manifest in various ways such as ulcers, inflammatory diseases, and cancer. Skin ulcers are open wounds that are usually caused by pressure, poor circulation, or injury. Inflammatory skin diseases, like atopic dermatitis (AD), psoriasis, vitiligo, etc., cause swelling, irritation, and redness of the skin. Melanoma refers to the abnormal growth of melanocytes that can lead to the development of malignant tumors (Anand et al., 2022). The treatment of skin problems often deals with symptom management and can be challenging due to the different nature of these conditions. A common obstacle in addressing skin issues involves the limited efficacy, the resistance to treatment and the potential side effects of drugs (Kim et al., 2021b; Yu et al., 2024). This can make it difficult to develop effective treatments without causing side effects or further damage to the skin. In this regard, drug delivery technologies have been developed to improve treatment outcomes in ways, including increasing therapeutic efficacy, decreasing toxicity, enhancing patient compliance, and enabling completely new medical treatments (Gao et al., 2023). Nowadays, a multitude of drug delivery systems have emerged, employing various technologies to facilitate more efficient, regulated, and precise delivery. The unique features of each system govern its specific release kinetics and mechanisms, which are influenced by the distinct physical, chemical, and structural properties that, in turn, impact their interactions with different pharmacological agents (Mattos et al., 2017). Recently, extensive research has been conducted on organic and inorganic nanomaterials to create specialized nanocarriers for drug transport (Watkins et al., 2015). Organic nanomaterials include natural or synthetic polymers and lipids. Among organic nanomaterials, polymeric nanocarriers are ideal for sustained therapeutic delivery with biocompatibility and biodegradability properties. A variety of synthetic polymers, such as polyethylene glycol (PEG), poly-L-lactic acid, polyvinyl alcohol, and poly (lactic-co-glycolic acid), along with natural polymers like chitosan and alginate, are extensively used in the construction of nanocarriers (Obeid et al., 2017). Likewise, dense lipid nanocarriers and phospholipids, including micelles and liposomes, are advantageous for drug delivery and targeting. Inorganic nanomaterials are made of carbon, silica, iron oxide, gold, and silver materials. These nanomaterials are mainly used for diagnosis and imaging, although it has been found that they have the potential for drug delivery. They offered benefits such as high drug-loading capacity, low toxicity, cost-effective laboratory production improved biocompatibility, and the ability to modify their surfaces (Baeza et al., 2017; Khafaji et al., 2019). Despite their potential, these carriers face clinical challenges due to their short half-life, limited ability to cross biological barriers, the possibility of interaction with biomolecules and accumulations in cells, and concerns about immunogenicity and toxicity (Min et al., 2015; Sukhanova et al., 2018). Exosome-based carriers, naturally derived from cells, offer promising solutions. Due to high stability, excellent biocompatibility, minimal immunogenicity, and the ability to be selectively loaded with therapeutic cargo, exosomes are ideal for drug delivery (Sharma and Mukhopadhyay, 2024). Notably, exosomes can cross biological barriers, reduce drug clearance, and exhibit lower toxicity compared to organic and inorganic nanoparticles. The lipid bilayer membrane of exosomes also protects against cargo degradation, making them an attractive option for drug delivery (Zeng et al., 2023; Yadav et al., 2024). Therefore, in this review, firstly exosomes are introduced as a natural carrier for loading different therapeutic agents. Then, various foreign cargoes delivered by exosomes will be discussed for treating skin diseases.

### 1.1 Exosome, delivery, and uptake

Exosomes are tiny vesicles surrounded by a lipid layer, typically ranging from 30 to 150 nm that release from different cells including mesenchymal stem cells (MSCs), immune cells, and cancer cells. They carry important molecules like proteins, nucleic acids, and lipids and play a crucial role in cell-to-cell communication (Keshtkar et al., 2018; Keshtkar et al., 2022b). Exosomes facilitate intracellular communication through several mechanisms: a) interaction between exosome membrane proteins and receptors on target cells, b) interaction between soluble fragments derived from exosome membrane proteins and cell surface receptors, and c) internalization of exosome contents by target cells (Keshtkar et al., 2022a; Keshtkar et al., 2022b). Recipient cells can internalize exosomes through various mechanisms, such as lipid raft-, caveolae-, and clathrin-dependent endocytosis, macropinocytosis, and phagocytosis, and some receptor binding such as integrins, scavenger receptors, complement receptors, and T-cell immunoglobulin- and mucin-domain-containing molecule-4. These processes allow exosomes to deliver cargo to recipient cells, enabling them to perform biological functions (Morelli et al., 2004; Miyanishi et al., 2007; Tian et al., 2010; Escrevente et al., 2011; Mittelbrunn and Sánchez-Madrid, 2012; Svensson et al., 2013; Prada and Meldolesi, 2016).

There are several isolation methods for exosomes, such as ultracentrifugation, density gradient centrifugation, ultrafiltration, size exclusion chromatography (SEC), immunoaffinity, and polymer precipitation (Kar et al., 2023). Ultracentrifugation has been introduced as a gold standard method. Although this method is cost-effective and ideal for large-scale samples, it is time-consuming, can damage exosomes, and results in low yield and modest purity. Density gradient centrifugation avoids exosomal damage and achieves high purity but is time-consuming, takes over 16 h, requires labor-intensive preliminary preparation, and results in minimal yield. Ultrafiltration is a simple and quick method that does not need special equipment, produces high-purity components with moderate yield, but can result in the loss of small-diameter exosomes due to membrane clogging. SEC provides highly specific exosome subtype isolation in just 0.3 h with high yield and purity, but special columns and packing are needed to address lipoprotein contamination. Immunoaffinity capture-based isolation allows for the specific targeting of exosome subtypes but requires more complex procedures and specific antibodies. Polymer precipitation is a straightforward method suitable for large-volume samples, yielding high amounts in 0.3–12 h. However, it may result in low purity due to contaminants from protein aggregates or residual polymers. Totally, the choice of method depends on factors such as sample volume, desired purity, and downstream application goals (Zhou et al., 2020; Kar et al., 2023).

### 1.2 Exosome manipulation and drug loading

**FIGURE 1:**
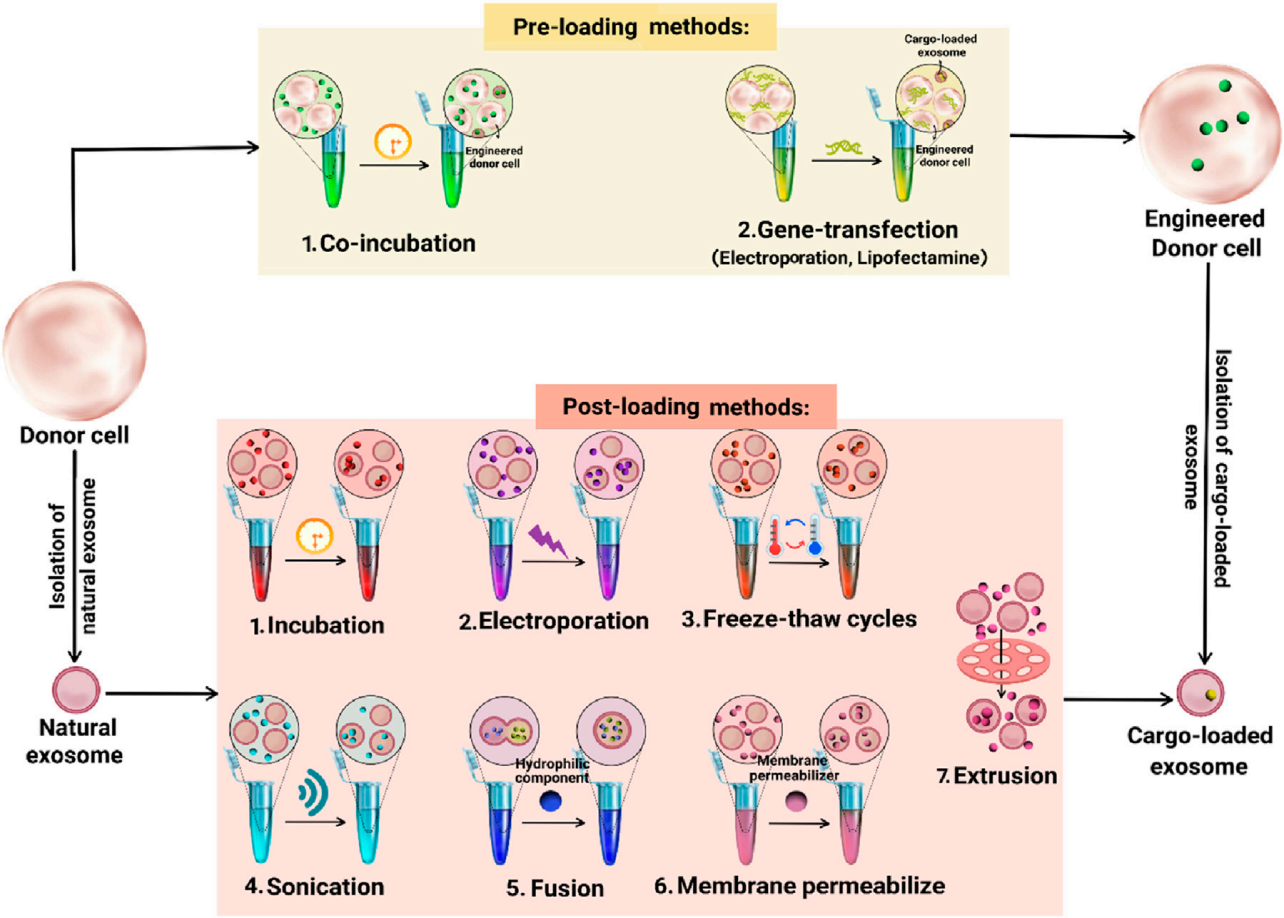
Drug-loading methods in exosome.

Various methods have been devised to incorporate therapeutic or diagnostic payloads such as chemical drugs, nucleic acids, proteins, peptides, and nanomaterials into exosomes. These techniques can be classified into pre-loading and post-loading methods (Figure 1) (Wang et al., 2021). In the pre-loading technique, cargos are loaded into donor cells before exosome isolation. This approach allows for the encapsulation of drugs within exosomes during their natural biogenesis. The process involves modifying donor cells by co-incubating them with the desired cargo or transfecting the target gene to release specific exosomes (Pascucci et al., 2014; Zhang H. et al., 2018). Notably, the pre-loading method ensures continuous production of cargo-loaded exosomes without compromising membrane integrity. However, it has limitations, including imprecise control over the amount of cargo loaded, low loading efficiency, potential gene expression changes in donor cells, and the risk of toxicity due to transfection agents (Liu et al., 2019; Xi et al., 2021; Kimiz-Gebologlu and Oncel, 2022).

The post-loading method involves loading drugs onto isolated natural exosomes. This approach has two distinct strategies: passive and active loading (Kimiz-Gebologlu and Oncel, 2022). Passive loading relies on the physical properties of the drug to passively diffuse into exosomes through co-incubation. Active loading employs various techniques to encapsulate the drug into exosomes including electroporation, sonication, fusion, freeze-thaw cycles, membrane permeabilizers, and extrusion (Xu et al., 2020; Xi et al., 2021). Electroporation uses a high-intensity electric field to create temporary holes in the exosome membrane, enabling drug entry. This method is effective for loading large molecules like microRNA (miRNA) and small interfering RNA (siRNA) but has a poor loading capacity due to RNA aggregation and stability issues (Sato et al., 2016; Akuma et al., 2019). Sonication disrupts the exosome membrane using ultrasonic waves, allowing hydrophobic drugs to pass through. This method maintains the integrity of exosome membranes and does not reduce membrane-bound protein or lipid content, but it can alter exosome viscosity (Kim et al., 2016; Huang et al., 2020). The fusion method combines exosomes and nanocomposites within a membrane structure, enhancing drug absorption, efficacy, and exocrine functions. This technique successfully enriches exosomes with hydrophilic components without compromising their function, significantly improving the cellular transport efficiency of chemotherapeutic agents (Akuma et al., 2019). Freeze-thaw cycles involve repeatedly freezing and thawing exosomes with drugs to improve encapsulation. This method has a lower drug loading capacity compared to sonication or extrusion and can cause exosome aggregation (Gurung et al., 2021; Kim et al., 2021a). Membrane permeabilizers like saponin interact with cholesterol to create pores in the membrane, improving the loading efficiency of enzymes like catalase into exosomes (Kim et al., 2016). The membrane extrusion method involves mixing exosomes with drug molecules and passing them through a nanofiltration membrane filter. This process creates fissures in the exosome membrane, allowing for drug loading. Researchers dissolve drugs in an appropriate solvent and slowly add them to exosomes. Devices like manual extruders or microgrinders are used to encapsulate drugs within exosomes (Liang et al., 2020). Generally, these techniques disrupt exosome membranes, enhancing drug encapsulation efficiency by up to 11 times. However, these methods can affect the exosome’s targeting properties and native structure (Akuma et al., 2019).

### 1.3 Routes of exosome administration

**FIGURE 2:**
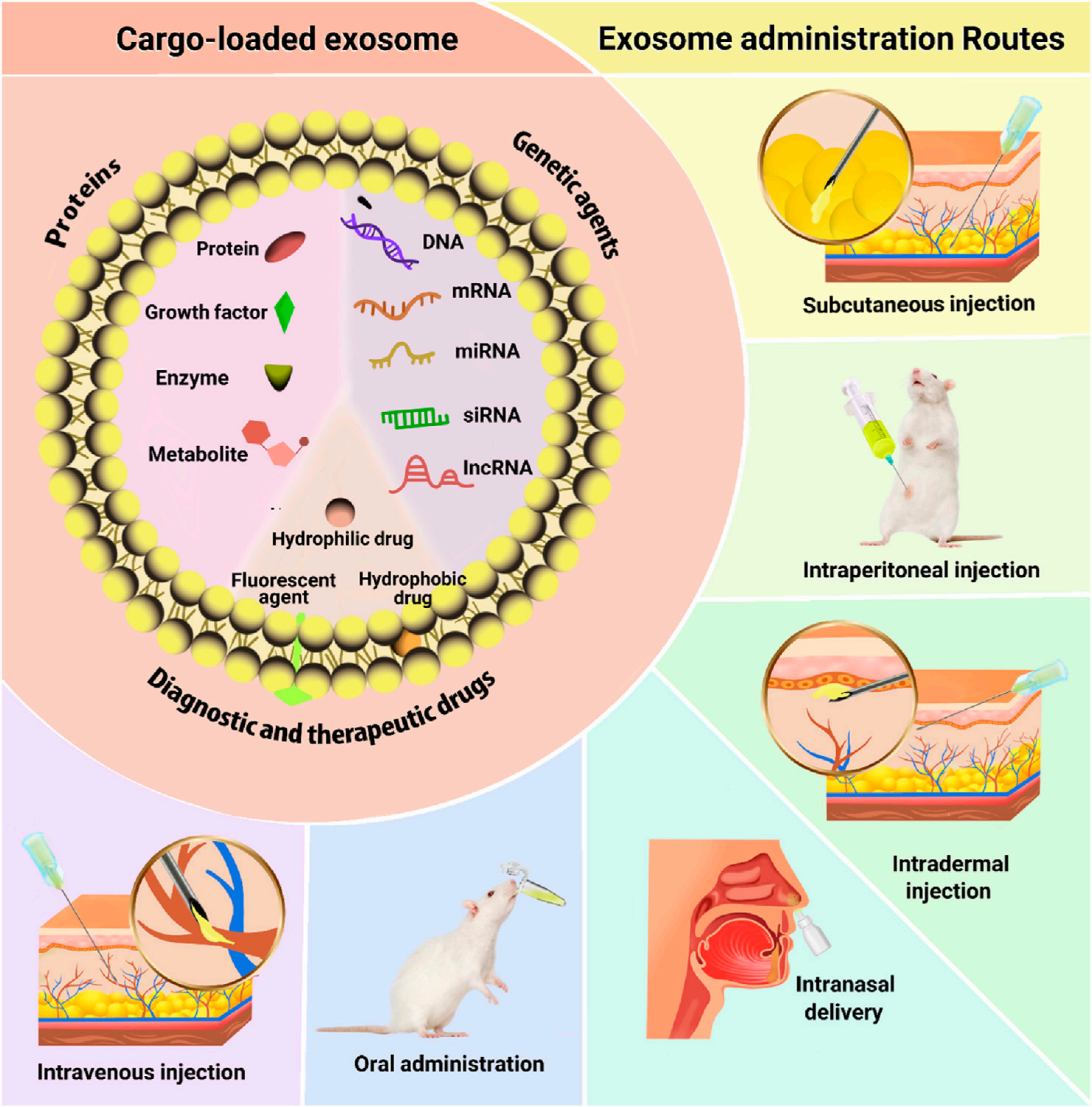
Exosome administration routes.

Exosome administration is a critical factor in therapeutic applications, with various routes offering distinct biodistribution and effects. Intravenous injection is common, enhancing treatments for heart, brain, and cancer conditions, despite rapid clearance by organs like the liver and lungs; modifications like PEGylation can extend circulation time (Kooijmans et al., 2016; Sun et al., 2018). Subcutaneous injections show promise in skin healing and malignancies, influencing immune cell behavior for faster recovery (Kim et al., 2019). Another method for administering exosomes into the body is through intradermal injection. This involves injecting exosomes into the dermis, which enhances wound closure rates, improves vascularity, and exhibits antitumor effects (Wang et al., 2019; Qiu et al., 2020). Intranasal delivery is effective for brain-targeted therapies, with studies showing protection against neurodegeneration and brain injuries (Zhuang et al., 2011; Liao et al., 2020). Intraperitoneal injections have yielded positive results in cancer and autoimmune disease models by modulating immune responses (Sun et al., 2010; Bellavia et al., 2017; Nojehdehi et al., 2018). Lastly, oral administration provides a non-invasive option, successfully delivering compounds like curcumin and inducing immune tolerance in autoimmune diseases (Figure 2) (Arntz et al., 2015; Aqil et al., 2017). This variability complicates the standardization of exosome delivery methods, making it difficult to achieve consistent therapeutic outcomes (Kang et al., 2021). Overall, the field needs further research to optimize dosing and evaluate the efficacy and safety of exosome-based treatments.

**FIGURE 3:**
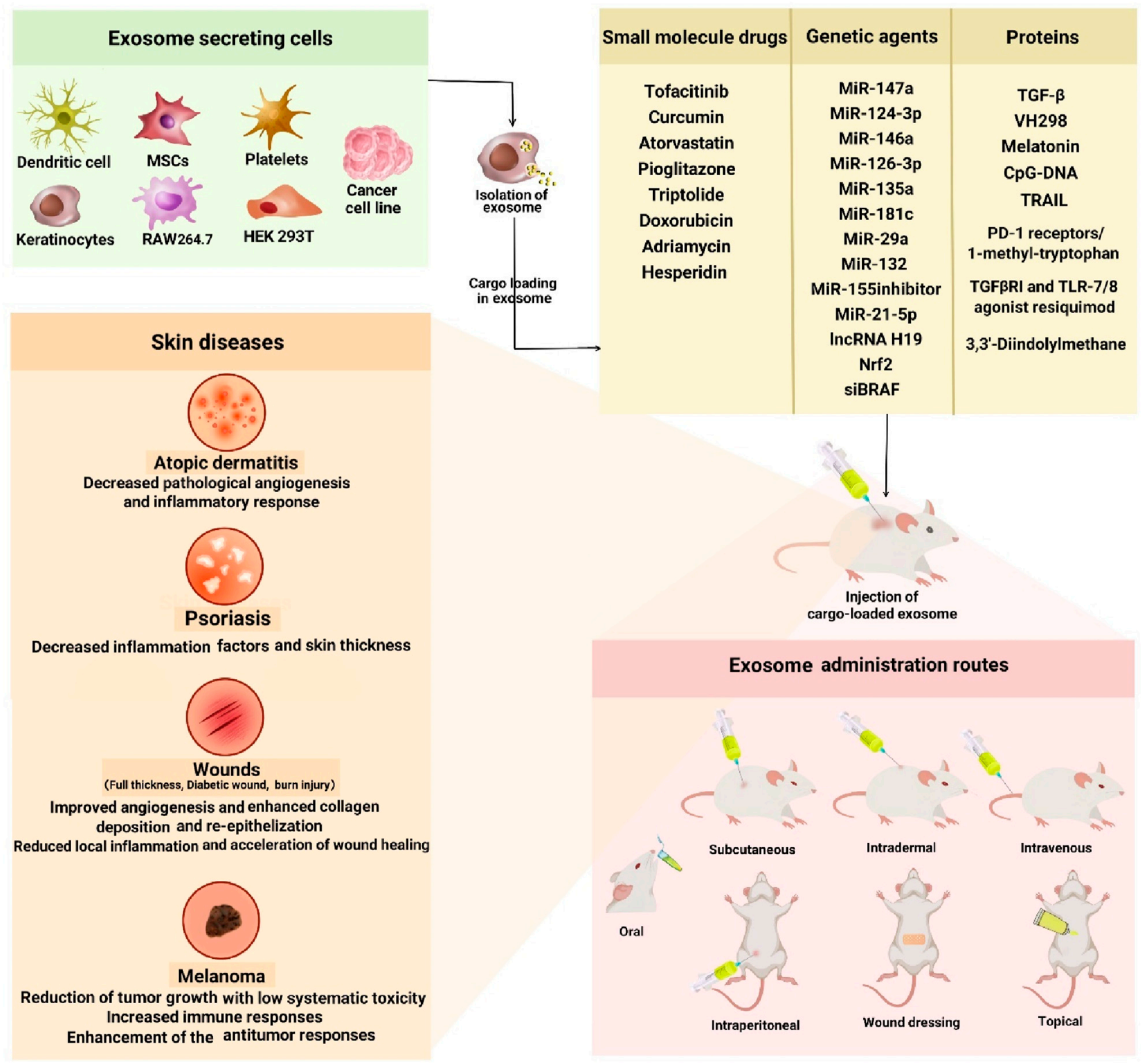
Application of exosomes for delivery of active biomolecules in the skin diseases. Exosomes released from different source of cells. Different kind of cargo loaded in isolated exosomes and injected to different type of skin diseases in animal models.

The potential of exosomes for drug delivery has garnered significant attention from the scientific community, highlighting their promise as a therapeutic strategy for various diseases, including cancer and neurodegenerative disorders (Gilligan and Dwyer, 2017; Das et al., 2018). In the following, the investigation of various cargoes loaded in exosomes for the treatment of skin diseases will be discussed and summarized in Figure 3.

## 2 Exosomes for delivering genetic agents in skin diseases

Gene therapy is a therapeutic approach based on various genetic materials, including messenger RNA (mRNA), miRNA, and DNA, for the treatment of various diseases including skin disorders. The delivery of genetic materials is challenging because the cell membrane prevents them from directly penetrating the cells in the body. In addition, genetic materials are very fragile and it is important to protect them from degradation by RNase in the extracellular fluid (Sarkar et al., 2020; Duan et al., 2021). Various RNA molecules can be loaded into exosomes and then delivered to specific cells or tissues using a range of biochemical or physical methods (Kim, 2020). Over the past years, notable advancements have been made in utilizing exosome-mediated gene therapy for the prevention and treatment of various diseases, including immune disorders, cancers, and infectious diseases. In continuation, the genetic agents loaded in exosomes and applied for skin diseases will be discussed (Table 1). AD is an inflammatory skin disease described by type 2 skin inflammation and a defective barrier (Shao et al., 2020). miRNA-147a, an anti-inflammatory micro-RNA in keratinocytes and endotheliocytes regulating inflammatory infiltration under pathological conditions, is a potential therapeutic agent for AD. According to the potential therapeutic effect of exosomes for AD and their carrier potential for gene delivery, Shi et al. innovatively developed a novel approach to alleviate AD by overexpressing miR-147a in adipose-derived stem cells (ADSCs). In this regard, they isolated exosomes to deliver miR-147a specifically to AD lesioned skin mice model. As a result, exosomes derived from miR-147a-overexpressing ADSCs extenuated pathological angiogenesis and inflammatory response during AD progression by targeting the VEGFA and MEF2A-TSLP axis (Shi et al., 2023).

**TABLE 1:** Application of exosomes as a delivery vehicle for genetic agents in skin diseases.

Exosome source	Cargo	Loading method	*In-vitro*/*In-vivo* model	Administration route	Result	Ref.
ADSCs	MiR-147a	Lipofectamine 3000	*In vivo*: Mice model of AD	—	Extenuated pathological angiogenesis and inflammatory response during AD progression	Shi et al. (2023)
Human keratinocytes	MiR-124-3p	Electroporation	*In vivo*: Mice model of psoriasis	Intradermally	Reduced skin thickness, as well as decreased epidermal thickness, proliferating cells, and the oxidative stress marker malondialdehyde	Longevity and Longevity (2023)
ADSCs	MiR-146a	Liposome	*In-vivo*: Full-thickness wounds of rat model	Injected into four sites of the wound	Promoted the neovascularization of full-thickness skin defects	Chen et al. (2023b)
ADSCs	MiR-126-3p	Liposome	*In-vivo* Full-thickness wounds of rat model	injected into the four sides of the wound	Increased collagen deposition and angiogenesis simultaneously in skin defects	Ma et al. (2022)
AMSCs	MiR-135a	Lipofectamine 2000	*In-vivo*: Full-thickness wound of rat model	Injected directly into the full-thickness wound margin	Promoted wound healing and epithelialization by promoting fibroblast migration	Gao et al. (2020)
UCMSCs	MiR-181c	Lipofectamine 2000	*In-vivo*: full-thickness burn injury rat model	Vein tail injection	Reduced burn-induced inflammation by downregulating targeting mRNA encoding TLR4 in macrophages	Li et al. (2016)
ADSCs	MiR-29a	Lipofectamine 2000	*In-vivo*: transdermal mice model For scar formation	Subcutaneous	Decreased scar formation by blocking the TGF-β/Smad3 pathway in fibroblasts	Yuan et al. (2021)
SMSCs	MiR-126	Lentivirus	*In-vivo*: Diabetic Wound of rat model	—	Improved angiogenesis and enhanced collagen maturation in ischemic diseases	Tao et al. (2017)
ADSCs	MiR-132	Lentivirus	*In-vivo*: the diabetic wound of mice model	Subcutaneous	Reduced local inflammation, promoting angiogenesis, and stimulating M2-macrophage polarization	Ge et al. (2023)
MSCs	MiR-155inhibitor	—	*In vivo*: diabetic wound mice model	—	Increased significantly collagen deposition, re-epithelization, and angiogenesis	Gondaliya et al. (2022)
ADSCs	MiR-21-5p	Electroporation	*In-vitro*: HaCaT cells *In vivo*: Diabetic wound of rat model	Applied evenly to the wound bed	Increased angiogenesis, vessel maturation, collagen remodeling, and re-epithelization in diabetic wounds. promoted proliferation and migration of keratinocytes through Wnt/β-catenin signaling *invitro*	Lv et al. (2020)
BMMSCs	lncRNA H19	Lipofectamine 2000	*In-vivo*: DFU mice model	Injected into the skin around the wound	Prevented the apoptosis and inflammation of fibroblasts by impairing PTEN inhibition, leading to the stimulated wound-healing process in DFU	Li et al. (2020a)
ADSCs	Nrf2	—	*In vivo*: DFU rat model	—	Improved wound healing by increasing the expression level of and VEGFR2 and decreasing the level of inflammatory cytokine IL-1b, Il-6, and TNF-a and oxidative stress proteins NOX1, NOX4, and ROS	Li et al. (2018)
mDC	siBRAF	electroporation	*In vitro*: B16F10 cells *In vivo*: melanoma mice model	subcutaneous	enhanced anti-malignant melanoma activity and a high level of safety *in vivo* Inhibited melanoma growth by promoting T cell proliferation *in vitro*	Lin et al. (2023)

Abbreviation: ADSCs, Adipose-derived stem cells; MSCs, Mesenchymal stem cells; AMSCs, amnion mesenchymal stem cells; UCMSCs, Umbilical Cord Mesenchymal Stem Cell; SMSCs, Synovium Mesenchymal Stem Cells; mDC, mature dendritic cell; HaCaT, human keratinocyte cell line; AD, Atopic dermatitis; siBRAF, BRAF, siRNA.

Psoriasis is a chronic inflammatory skin disease described by abnormal differentiation and reduction of keratinocytes (Shao et al., 2020). MiR-124-3p can inhibit psoriasis-like skin inflammation by targeting STAT3 mRNA in keratinocytes. In the study of Liu et al., exosomes were isolated from human keratinocytes and miR-124-3p was transfected by electroporation to obtain Exo-miR-124-3p. Then, the injection was intradermally performed on the back skin of psoriasis mice. The results showed that miR-124-3p alleviated skin inflammation and reduced skin thickness, as well as decreased epidermal thickness, proliferating cells, and the oxidative stress marker malondialdehyde (Longevity and Longevity, 2023).

MiR-146a acts as a critical molecular brake on aberrant inflammation targets mRNA encoding caspase recruitment domain protein 10 (CARD10) and regulates angiogenesis. MiR-146a also targets mRNA encoding CARD10 and regulates angiogenesis. Chen et al. transfected miR-146a to human ADSCs by liposomes. They extracted exosomes from miR-146a-modified ADSCs. Exosomes were injected into rats at four sites around skin defects. They showed that miR-146a promoted the neovascularization of full-thickness wounds in rats (Chen M. et al., 2023).

MiR-126-3p directly encodes phosphoinositide-3-kinase (PI3K) regulatory subunit 2 mRNA in skin fibroblasts and vascular endotheliocytes. Ma et al. transfected miR-126-3p inhibitor to human ADSCs cells by liposomes. Then, Exo-miR-126-3p inhibitors were isolated from the culture supernatant after 24 h and injected into the four sides of the full-thickness wound in rats. They found that topical treatment of wounds increased collagen deposition and angiogenesis simultaneously in skin defects (Ma et al., 2022).

MiR-135a targets mRNA encoding large tumor suppressor factor 2 (LATS2) in fibroblasts, promoting local fibroblast migration into wounds. Gao et al. transfected a miR-135a plasmid into human amnion MSCs(hAMSCs) by liposomes. Then, Exo-miR-135a was isolated from the culture supernatant after 48 h. After that, the Exo-miR-135a was mixed with collagen-I, and injected directly into the full-thickness wound margin of rats. After 5 days of treatment, rats receiving Exo-miR-135a showed the fastest wound healing. They found that miR-135a promotes wound healing and epithelialization by promoting fibroblast migration (Gao et al., 2020).

MiR-181c in the exosomes demonstrated a pivotal role in regulating inflammation. Li et al. transfected miR-181c into human umbilical cord MSCs (hUCMSCs) by liposomes, and Exo-miR-181c was obtained from the culture supernatant and applied for the treatment of the full-thickness burn injury rat model. They found that burn injury significantly increased the inflammatory reaction of rats. These results indicated that miR-181c can reduce burn-induced inflammation by downregulating mRNA encoding TLR4 in macrophages (Li et al., 2016).

The expression level of miR-29a in human burn scars and hypertrophic scar fibroblasts is significantly reduced compared to normal tissue and fibroblasts. Yuan et al. transfected miR-29a into hADSCs by liposomes. Exosomes derived from miR-29a-modified hADSCs were extracted and injected subcutaneously into a transdermal mice model to evaluate scar formation. They found that transforming growth factor beta (TGF-β) was the target of miR-29a and inhibited scar hyperplasia by the TGF-β/Smad3 pathway in fibroblasts (Yuan et al., 2021).

Tao et al. investigated the therapeutic effect of overexpressing miR-126 in exosomes derived from synovial MSCs (SMSCs) on diabetic wounds in rats. In this study, SMSCs were transfected with miR-126 by lentivirus, then exosomes were extracted from SMSC-126 and injected into a diabetic rat model. They showed that exosomes derived from SMSC-126 improved angiogenesis in human dermal fibroblasts and human dermal microvascular endothelial cells (HDMEC-1) and enhanced collagen maturation in ischemic diseases through PI3K/AKT and MAPK/ERK (Tao et al., 2017).

MiR-132 targets the TGF-β, PI3K/AKT, and Hippo pathways, thereby promoting proliferation and migration. Ge et al. loaded lentivirus carrying miR-132 into ADSCs. Exosomes derived from miR-132-overexpressing ADSCs (miR-132-exo) were harvested from the supernatant and injected subcutaneously in four points around the wound in diabetic mice. The results showed miR-132-exo accelerates diabetic wound healing, by reducing local inflammation, promoting angiogenesis, and stimulating M2-macrophage polarization mediated by the NF-κB signaling pathway (Ge et al., 2023). In another study, Gondeliya et al. investigated the effect of directly loaded miR-155 inhibitor into MSC-Exosomes on a diabetic mice model. They showed that the treatment of wounds by miR-155 inhibitor-exosomes increased significantly collagen deposition, re-epithelization, and angiogenesis (Gondaliya et al., 2022).

Lv et al. loaded miR-21-5p into human ADSCs by electroporation. Then they extracted the exosomes containing miR-21-5p from ADSCs and applied them evenly to a wound bed in a diabetic rat model. The modified exosome increased angiogenesis, vessel maturation, collagen remodeling, and re-epithelization in diabetic wounds. *In vitro* study on a human keratinocyte cell line (HaCaT) loaded with miR-21-5p-exosomes showed that the engineered exosome promoted the proliferation and migration of keratinocytes through Wnt/β-catenin signaling (Lv et al., 2020).

lncRNA H19 targets phosphatase and PTEN and binds to microRNA-152-3p. Li et al. loaded lncRNA H19 in mice bone marrow MSCs (BMMSCs). Exosomes containing lncRNA H19 were extracted from BMMSCs and injected into the surrounding tissues of the wound, in a mouse model of diabetic foot ulcer (DFU). They found that MSC-derived exosomal lncRNA H19 prevented the apoptosis and inflammation of the fibroblasts by impairing miR-152-3p-mediated PTEN inhibition, leading to the stimulated wound-healing process in DFU (Li B. et al., 2020).

Nrf2 is involved in cell migration, proliferation, apoptosis, and differentiation. Li et al. transfected Nrf2 into human ADSCs. Exosomes derived from Nrf2 overexpressing ADSCs were isolated and used to treat the DFU rat model. They found that increasing the expression level of VEGFR2 and decreasing the level of inflammatory cytokine interleukin (IL)-1b, Il-6, and TNF-α and oxidative stress proteins NOX1, NOX4, and ROS resulted in improved wound healing in DFU rat model (Li et al., 2018).

In melanoma, a kind of skin cancer, BRAF siRNA (siBRAF) can effectively inhibit the invasion and metastasis of malignant cells. Lin et al. used mature dendritic cell (mDC) exosomes (mDexos) as vectors to carry siBRAF by electroporation. A melanoma mice model was established and treated by siBRAF-mDexos via subcutaneous injection. They showed significantly enhanced anti-malignant melanoma activity and a high level of safety *in vivo*. Moreover, siBRAF-mDexos were examined on B16-F10 cells *in vitro* and found to significantly inhibit melanoma growth by promoting T cell proliferation (Lin et al., 2023).

## 3 Exosomes for small molecule drug delivery in skin diseases

Despite the excellent therapeutic benefits of small molecule drugs in skin diseases, their low solubility, *in vivo* instability, short half-life, and poor oral bioavailability have limited their potential as a clinical therapeutic (Yadav et al., 2024). Exosomes hold great promise as an effective drug delivery vehicle in the treatment of skin diseases (Sharma and Mukhopadhyay, 2024). They can deliver desired therapeutic payloads with enhanced bioavailability, improved drug stability, reduced drug dose, be specific-targeted, and pass across many physical barriers through surface modification (Ortega et al., 2021; Joorabloo and Liu, 2023). These features render exosomes highly suitable for the treatment of various skin diseases. Exosomes have been demonstrated to promote angiogenesis and upregulate anti-inflammatory factors; thus, exosome-based drug delivery systems have been extensively studied in skin inflammation, psoriasis, wound healing, and melanoma (Table 2) (Hou et al., 2022; Zhao et al., 2023; Jonoush et al., 2024; Shi et al., 2024).

**TABLE 2:** Application of exosomes for small molecule drug delivery in skin diseases.

Source of exosomes	Loaded cargo	Loading method	*In vitro*/*in vivo* models	Administration routs	Results	Ref
A-431	TFC	Incubation, freeze-thaw, probe sonication, and ultrasonic bath	*In vivo*: imiquimod-induced psoriasis mice model	Topical	Less cytotoxicity *in vitro*, higher suppression of the expression of TNFα, IL-23, IL-6, and IL-15 genes, and better therapeutic effect *in vivo* compared to free drug	Dehghani et al. (2024)
Mice J774A.1	Albumin and curcumin	Mild probe sonication	*In vivo*: imiquimod-induced psoriasis-like and LPS-triggered inflammation in rat and mice model	Applied manually and held in place for 10 min using a finger	Improvement of stability *in vitro*, reduction of inflammation protein compared to free drug	Yerneni et al. (2022)
RAW264.7	Curcumin	—	*In vitro*: HUVEC *In vivo*: diabetic wound rat model	Subcutaneous	Promotion of proliferation, migration, and angiogenesis of HUVECs and acceleration of wound healing *in vivo*	Li et al. (2023)
BMMSCs	ATV-pre treated	—	*In vitro*: HUVEC *In vivo*: STZ-induced diabetic rats	Subcutaneous	Improvement of proliferation, migration, and VEGF expression of HUVEC and acceleration of wound regeneration *in vivo*	Yu et al. (2020)
BMMSCs	PGZ-pretreated	—	*In vitro*: HUVEC *In vivo*: STZ-induced diabetic rats	Subcutaneous	Enhancement of the migration, tube formation, and VEGF expression in HUVEC and enhancement of the therapeutic efficacy of MSCs-derived exosomes and acceleration of diabetic wound healing *in vivo*	Hu et al. (2021)
Bovine milk	Hesperidin	Probe sonication	*In vitro*: B16F10 *In vivo*: B16F10-induced melanoma in Swiss mice	Oral and intraperitoneal	Enhancement of the half-life and therapeutic efficacy of hesperidin -loaded exosomes in both *in vitro* and *in vivo*	Kumar et al. (2024)
-	Adriamycin	Incubation	*In vitro*: A375 or WM266-4 cells *In vivo*: mice model of melanoma	Intravenous	Exhibition of good biocompatibility *in vitro*, accumulation in the tumor more than free exosomes, and showed the lowest tumor weight *in vivo*	Chen et al. (2023a)
UCMSCs	TP	Incubation	*In vitro*: A375 and HaCaT cells *In vivo*: mice model of melanoma	Intravenous	Inhibition of proliferation, invasion, and apoptosis promotion *in vitro*, excellent melanoma-targeting ability, and prolonged half-life of TP *in vivo*	Gu et al. (2022)
B16.F10	Dox	Incubation	*In vitro*: B16.F10 *In vivo*: the B16.F10 melanoma-bearing mice	Intravenous	Exhibition of higher inhibitory effects on the proliferation *in vitro* and a stronger antitumor efficacy *in vivo*	Patras et al. (2022)
B16.F10	Dox	Incubation	*In vitro*: B16F10 *In vivo*: the B16.F10 melanoma-bearing mice	Intravenous	Exhibition of strong antiproliferative effect on tumor cells co-cultured with TAMs by administration of IL-13-LCL-SIM and PEG-EV-Dox	Negrea et al. (2022)
B16.F10	Dox	Sonication and Incubation	*In vitro*: B16F10 *In vivo*: the B16.F10 melanoma-bearing mice	Intravenous	Enhancement of the therapeutic activity of Dox *in vitro* and suppression of tumor development with low systematic toxicity *in vivo*	Kang et al. (2020)
RAW264.7	TP	Mild ultrasonic	*In vitro*: A375 *In vivo*: the B16.F10 melanoma-bearing mice	Intravenous	Enhancement of the targetability of exosomes and induced tumor cell apoptosis by specifically binding to DR5 in melanoma mice models	Jiang et al. (2021)

Abbreviations: TFC, tofacitinib; TNFα, Tumor Necrosis Factor-Alpha; IL-23, Interleukin 23; IL-6, Interleukin 6; IL-15, Interleukin 15; LPS, lipopolysaccharides; HUVEC, human umbilical vein endothelial cells; BMMSCs, Bone Marrow Mesenchymal Stromal Cells; VEGF, vascular endothelial growth factor; MSCs, Mesenchymal Stem Cells; ATV, atorvastatin; STZ, streptozotocin; PGZ, pioglitazone; hUCMSCs, Human Umbilical Cord Mesenchymal Stem Cells; TP, triptolide; Dox, Doxorubicin; TAMs, Tumor-Associated Macrophages; IL-13-LCL-SIM, Simvastatin incorporated in Interleukin-13-functionalized Long-Circulating Liposomes; PEG-EV, PEG-coated Extracellular Vesicles; DR5, Death Receptor 5.

Tofacitinib (TFC), as an immunosuppressive drug, is an oral inhibitor of JAK1 and JAK3 leads to a multi-tiered intervention in psoriasis, with a direct impact on dysregulated keratinocytes, declines the expression of the proinflammatory cytokine, and normalizes the IL-23/Th17 axis (Tian et al., 2019). Dehghani et al. successfully designed TFC-loaded A341-derived exosomes to reduce inflammation of psoriasis in the imiquimod-induced psoriasis mice model. Moreover, a significant reduction in the cytotoxicity of TFC-loaded exosomes compared to free TFC was observed probably due to the slow release of TFC. After 24 h, topical TFC-loaded exosomes augmented the expression of the inflammatory cytokine gene involved in psoriasis and exhibited the best therapeutic effect in the imiquimod-induced psoriasis mice model (Dehghani et al., 2024).

In another study, Yerneni et al. introduced a skin-targeted delivery system based on curcumin-albumin-exosomes (CA-exosomes) using dissolvable microneedle arrays (dMNAs) to explore the synergistic impact of these drugs to suppress imiquimod-induced psoriasis- model and LPS-triggered inflammation *in vivo*. Compared with curcumin and curcumin-exosomes, dMNAs-delivered CA-exosomes showed the highest storage stability *in vitro*, suggesting the role of exosomes and dMNAs protection. Approximately 5 days after the skin inflammation in rat and mouse models, the dMNA application of CA-exosomes decreased the thickness of the skin, blocked inflammation, and downregulated inflammatory protein (Yerneni et al., 2022).

Curcumin is a natural polyphenol compound with an antiseptic, antioxidant, and anti-inflammatory activity that has the potential to treat various inflammatory diseases (Cozmin et al., 2024). In this regard, Li et al. loaded curcumin into exosomes derived from macrophages and injected subcutaneously around the wound in a diabetic wound rat model. The loaded exosomes facilitated the proliferation, migration, and angiogenesis of human umbilical vein endothelial cells (HUVECs), inhibited inflammation, and reduced reactive oxygen species production. *In vivo* investigations revealed that curcumin-loaded exosomes increased re-epithelialization and collagen deposition, produced mature granulation tissue, and upregulated the expression of wound healing-related molecules compared with the control group (Li et al., 2023).

Moreover, exosome pre-treatment with various drugs like pioglitazone (PGZ) and atorvastatin (ATV) promoted chronic wound healing (Liu et al., 2020; Yu et al., 2020; Hu et al., 2021). ATV, a commonly used lipophilic statin for oral lipid-lowering, has been reported to have numerous effects, including promoting angiogenesis, increasing VEGF secretion, and facilitating wound healing (El-Okaily et al., 2023). Yu et al. asserted that exosomes derived from ATV-treated BMMSCs (ATV-Exosome) provide better support for the proliferation, migration, and VEGF expression of HUVEC via AKT/iNOS signaling pathway compared to the use of non-pretreated BMMSCs. Injection of ATV-Exosome subcutaneously accelerated wound regeneration in streptozotocin (STZ)-induced diabetic rats by enhanced blood vessel formation compared to the controls (Yu et al., 2020). Recently, the findings showcased the potential protective role of PGZ, as an anti-diabetic medication, in inflammation and oxidative stress to facilitate tissue renovation processes. They reported the superior effects of PGZ-pretreated MSCs-derived exosomes (PGZ-Exosome) on the biological functions of HUVECs, such as tube formation, migration, VEGF expression, and wound regeneration *in vitro* by targeting the PI3K/AKT/eNOS pathway. Their findings indicated that subcutaneous injection of PGZ-Exosome in STZ-diabetic rats leads to regenerative events including VEGF and CD31 expression, ECM remodeling, and collagen deposition, suggesting angiogenesis in diabetic wound healing (Hu et al., 2021).

Hesperidin, a flavonoid found in citrus fruits, reveals various pharmacological activities, including anti-microbial, anti-cancer, anti-inflammation via apoptosis pathways, and modulation of numerous anti-inflammatory factors (Talebi et al., 2024). To this aim, Kumar et al. isolated exosomes from bovine milk and developed hesperidin-loaded exosomes in the B16F10-induced melanoma mice model. *In vitro* investigations on B16F10 cell lines verified higher cytotoxicity of hesperidin-loaded exosomes than free hesperidin, as supported by enhanced cellular uptake. Oral pharmacokinetics investigations showed a significant (approximately 2.5-fold) increase in half-life and oral bioavailability of hesperidin upon loading into exosomes. Hesperidin-loaded exosome intraperitoneally showed excellent anti-cancer activity compared to free hesperidin after oral administration *in vivo*. Notably, no histological, hematological, or biochemical toxicities were detected in hesperidin-loaded exosome-treated tumor-bearing mice (Kumar et al., 2024).

In addition, modification of exosomes with a variety of targeting ligands, stimuli-responsive, and immune evasion elements have been addressed to improve their drug delivery capability (Zhu et al., 2021; Fan et al., 2022). As a traditional chemotherapy drug for treating many cancers, adriamycin (also known as Dox) is limited in treating melanoma cancer stem cells (CSCs) due to adverse effects and poor solubility (Abdel-Megeed et al., 2024). To target CD20^+^ melanoma CSCs, Chen et al. developed anti-CD20 aptamer-modified exosomes (ACEXO) loaded with adriamycin. They incorporated cyclic RGD (cRGD) peptide on the exosome membrane by post-insertion method and conjugated CD20 aptamer to exosome. The modified exosome differed in cytotoxicity assay, with ACEXO killing only CD20^+^ melanoma cells while showing proper biocompatibility with 3T3 and 293T cells. By utilizing anti-CD20 aptamer, they effectively reduced the number of tumor spheres of WM266-4 or A375 cells compared to untreated controls or ACEXO-treated groups. Interestingly, *in vivo* investigations on a mice tumor model indicated tumor-targeting capabilities of the intravenously administered exosome and the lowest tumor weight, implying the substantial potential of this exosome in the selective suppression of human CD20^+^ melanoma stem cells (Chen H. et al., 2023).

Triptolide (TP) has been established to suppress multiple tumors, such as melanoma by regulating autophagy, apoptosis, and angiogenesis (Feng et al., 2024). Gu et al. designed exosomes with conjugated cRGD motif, as the αvβ_3_ integrin receptor overexpressed on the tumor cells and explored their potential to promote targeted delivery of TP in melanoma-bearing mice intravenously. The synthesized cRGD-Exosome/TP remarkably suppressed invasion, proliferation, and apoptosis promotion *in vitro*. Moreover, the *in vivo* pharmacokinetics investigations showed that cRGD-Exosome/TP possessed a superior melanoma-targeting capability and sustained the half-life of TP. Finally, *in vivo* antitumor results revealed that cRGD-Exosome/TP significantly suppressed tumor development and extended the survival time of tumor-bearing mice (Gu et al., 2022).

Recent studies have reported the utility of functionalized exosomes based on PEG modification in the targeted delivery of Dox in B16.F10 murine melanoma. Importantly, PEGylation of exosomes offers numerous benefits such as improving systemic circulation time, solubility, stability, and permeability, however reducing immunogenicity (Gao et al., 2024). In this context, Patras et al. reported much higher inhibitory effects of Dox-loaded PEGylated exosomes (PEG-EXO-DOX) intravenously injection on the proliferation of B16F10- induced mice melanoma model. Moreover, the functionalized exosomes enhanced antitumor activity of PEG-EXO-DOX in B16.F10 murine melanoma models as well as increased BAX expression and reduced NF-κB activation compared to liposomal Dox, suggesting better prognosis of exosome-based therapy than liposomal based treatment (Patras et al., 2022). In another study, Negera et al. explored the combinational therapy with simvastatin incorporated in IL-13-functionalized long-circulating liposomes (IL-13-LCL-SIM) based on PEG-EXO-Dox can selectively target both tumor-associated macrophages and melanoma cells. Findings showed that sequential intravenous administration of IL-13-LCL-SIM and PEG-EXO-Dox had the most powerful antiproliferative outcome on melanoma cells (Negrea et al., 2022).

Kang et al. engineered a more complex platform focusing on functionalized exosome using active targeting modality consist of membrane anchor (BODIPY)-spacer (PEG)-targeting ligand (RGD), named ASL conjugate, in B16F10 melanoma tumors. The Dox-loaded ASL exosomes (dAExosome) significantly inhibited B16F10 melanoma growth. In the mice model of cancer, intravenous injection of dAExosome attained tumor-targeted imaging and effectively inhibited the tumor development with low systematic toxicity as evidenced by body weight and tumor volume (Kang et al., 2020).

Furthermore, the integration of tumor necrosis factor-related apoptosis-inducing ligand (TRAIL) into exosomes can prompt cancer cell apoptosis by binding to death receptor5 (DR5) overexpressed in cancer cells. Jiang et al. employed a targeted delivery system based on TRAIL-engineered exosomes (TRAIL-exosome) to load TP to treat malignant melanoma. TP loading into the TRAIL-exosome system improved tumor targetability and cellular uptake while inhibiting proliferation, invasion, and migration, and inducing apoptosis of A375 cells via activating the intrinsic mitochondrial and the extrinsic TRAIL pathways *in vitro*. Furthermore, *in vivo* investigations on a melanoma nude mice model indicated that injection of TRAIL-exosome-TP intravenously suppresses tumor progression capabilities, implying the promising potential of this delivery system in the treatment of malignant melanoma (Jiang et al., 2021).

## 4 Exosomes for delivery of proteins and growth factors in skin diseases

Deficiency of certain proteins or growth factors is an important cause of many clinical problems. Exosomes are potential natural candidates for delivering bioactive molecules such as proteins, growth factors, and peptides. There are limited investigations on applying loaded exosomes with the mentioned substances in skin diseases (Table 3). The controlled release of bioactive molecules from exosomes has been introduced as a promising strategy in wound healing. Shi et al. developed a TGF-β loaded clinical-grade platelets exosome product incorporated in an injectable surgical fibrin sealant. They found that topical treatment of ischemic wounds of rabbits with this construct leads to regenerative events including matrix remodeling, cell proliferation, and re-acquisition of accessory structures of the skin (Shi et al., 2021).

**TABLE 3:** Application of exosomes for delivery of proteins and growth factors in skin diseases.

Source of exosomes	Loaded cargo	Loading method	*In vitro*/*in vivo* model	Administration route	Result	Ref.
Platelets	TGF-β	Incubation	*In vivo*: Ischemic full-thickness wounds of rabbits	Topical	Regenerative events including matrix remodeling, cell proliferation, and reacquisition of accessory structures of the skin	Shi et al. (2021)
Epidermal stem cells	VH298	Incubation	*In vivo*: Diabetic wound of mice	Wound dressing	Appropriate mechanical and biological characteristics	Wang et al. (2022)
Pretreatment of BMMSCs	Melatonin	—	*In vivo*: Diabetic wound of mice	Multisite subcutaneous injection	Elevated ratio of M2 to M1 macrophage polarization and extinguished the inflammatory response	Liu et al. (2020)
Pretreatment of UCMSCs	3,3′-Diindolylmethane	—	*In vivo*: a deep second-degree burn injury rat model	Subcutaneous	Upregulation of Wnt11 which led to β-catenin activation and triggered therapeutic effects *in vivo*	Shi et al. (2017)
RAW 264.7	TGFβRI kinase inhibitor SD-208 and a TLR-7/8 agonist resiquimod (R848)	Sonication and electroporation	*In vitro*: B16F10 cells isolated from the skin tissue of mice *In vivo*: the B16F10 tumor xenograft model	Tail-vein injection	Reduction of the migration of B16F10 cells and stimulation of the release of proinflammatory cytokines from macrophages and dendritic cells Reduction of tumor growth and promotion of survival rate	Lee et al. (2022)
Tumor cell	CpG-DNA	Incubation	*In vivo*: mice model of melanoma	Intradermally	Activation of dendritic cells Increase in immune responses	Matsumoto et al. (2019)
B16BL6	CpG-DNA	Incubation	*In vivo*: mice model of melanoma	Intradermally	Activation of DC2.4 cells, promotion of tumor antigen presentation capacity, and antitumor effects	Morishita et al. (2016)
HEK 293T cells	PD-1 receptors/1-methyl-tryptophan	Incubation	*Invitro*: B16F10 cells *In vivo*: Mice model of melanoma	Tail-vein injection	Enhancement of the antitumor responses against the melanoma tumor and ability to disrupt the immune tolerance	Zhang et al. (2018b)
Pretreatment of BMMSCs	TRAIL	Incubation	*In vitro*: B16F0 cell lines *In vivo*: Mice model of melanoma	Injection into the left flank	Massive necrosis in the cancer cells, inhibition of tumor progression	Shamili et al. (2018)

Abbreviations: TGF-β, transforming growth factor beta; VH298, (Von Hippel-Lindau, the E3 ligase); BMMSCs, Bone Marrow Mesenchymal Stem Cells; UCMSCs, Umbilical Cord-Derived Mesenchymal Stem Cells; Wnt11, Wnt Family Member 11; TGFβRI, Transforming growth factor-β receptor I; TLR, Toll-Like Receptor; DC: dendritic cells; PD-1, Programmed Cell Death 1; TRAIL, Tumor Necrosis Factor-Related Apoptosis-Inducing Ligand.

The lack of angiogenesis has been recognized as the main cause of diabetic wound healing complications. A recent study demonstrated that delivering VH298 into endothelial cells by exosomes derived from epidermal stem cells improves the function of the human HUVEC *in vitro* (Wang et al., 2022). VH298 is a small molecule designed by Frost et al. for stabilizing hypoxia-inducible factor-1α (HIF-1α) (Frost et al., 2016). Applying a mixture of gelatin methacrylate (GelMA) and VH298 loaded in exosomes on diabetic wounds showed appropriate mechanical and biological characteristics. This study suggested this construct is a beneficial wound dressing (Wang et al., 2022).

Moreover, exosome pre-treatment with small molecules like melatonin and 3,3′-Diindolylmethane could effectively ameliorate chronic and acute wound healing (Shi et al., 2017; Liu et al., 2020). In this regard, multisite subcutaneous injection of melatonin-pretreated MSC-derived exosomes represented beneficial effects on diabetic wound healing. Investigation of the underlying mechanism revealed the role of the PTEN/AKT signaling pathway in the elevated ratio of M2 to M1 macrophage polarization and subsequently extinguished the inflammatory response (Liu et al., 2020). Additionally, the subcutaneous administration of 3,3′-Diindolylmethane-pretreated hUCMSCs exosomes in a deep second-degree burn injury rat model improved the healing capacity of cells. This study indicated that these exosomes upregulated Wnt11, leading to β-catenin activation and triggering therapeutic effects *in vivo* (Shi et al., 2017). These reports showed that the prime of MSCs with these bioactive molecules results in the release of exosomes with elevated wound healing ability.

In melanoma, the RAW 264.7-derived exosomes were loaded by the transforming growth factor-β receptor I (TGFβRI) kinase inhibitor SD-208 and a toll-like receptor (TLR)-7/8 agonist resiquimod (R848). Findings showed that the exosomes loaded with SD-208 decreased the migration of B16F10 cells isolated from the skin tissue of a mouse with melanoma. Moreover, the exosomes loaded with R848 stimulated the release of proinflammatory cytokines from macrophages and dendritic cells. The *in vivo* study revealed that the combination of dual-loaded exosomes through the tail-vein injection resulted in the reduction of tumor growth and promotion of survival rate in the B16F10 tumor xenograft model (Lee et al., 2022). In addition, immunostimulatory CpG-DNA loaded in exosomes seems to be an effective strategy in the immunotherapy of melanoma. Researchers constructed a CpG-DNA anchored tumor cell-derived exosomes in this regard. They found that this structure effectively activated dendritic cells *in vitro* and increased immune responses when injected intradermally into the mice (Matsumoto et al., 2019). Furthermore, intradermally murine melanoma B16BL6-derived exosomes along with CpG-DNA could efficiently activate DC2.4 cells and promote tumor antigen presentation capacity. The *in vivo* administration of this engineered construct in B16BL6 tumor-bearing mice exhibited antitumor effects (Morishita et al., 2016).

In utilizing exosomes in cancer therapy, the engineered cellular nanovesicles presenting programmed cell death protein 1 (PD-1) receptors were applied. In this regard, B16F10 cells were transfected with EGFP-PD-L1 plasmid and then incubated with PD-1 nanovesicles. The administration of the produced nanovesicles through the tail-vein of mice model of melanoma indicated that this strategy enhanced antitumor responses against the melanoma tumor by disrupting the PD-1/PD-L1 inhibitory axis. On the other hand, by loading 1-methyl-tryptophan, an inhibitor of indoleamine 2,3-dioxygenase, into these engineered cellular nanovesicles their ability was enhanced to disrupt the immune tolerance pathway (Zhang X. et al., 2018). In cancer therapy, researchers also examined the effect of the exosomes derived from TRAIL--engineered MSCs in tumor activity in a melanoma model. They found that injection of these exosomes into the left flank of the mice leads to massive necrosis in the cancer cells which can inhibit tumor progression (Shamili et al., 2018).

## 5 Challenges, future ahead, and conclusion

Skin diseases include a wide range of diseases from self-limited and benign to malignant and divesting conditions. Due to the complex conditions of each disease, dermatologists face different challenges for targeted treatment (Shao et al., 2020; Kim et al., 2021b; Yu et al., 2024). Exosomes have unique properties like stability, minimal immune response, and strong biocompatibility, making them excellent vehicles for drug delivery. These attributes allow exosomes to efficiently penetrate target cells, evading detection and clearance by the immune system, and thus facilitating the delivery of foreign small molecules, protein, and nucleic acid medications to specific cells (Zeng et al., 2023). Exosomes can enhance drug efficacy by enhancing drug solubility and facilitating the simultaneous delivery of multiple drugs, thanks to their lipid bilayer structure with both lipophilic and hydrophilic properties (Zeng et al., 2023). The studies reviewed in this manuscript demonstrated that the exogenous cargoes loaded and transferred through exosomes can help in the treatment of many skin problems such as AD, psoriasis, skin wounds, and melanoma. A wide range of drugs from microRNAs to small molecules drugs such as curcumin, tofacitinib, triptolide, etc. to proteins such as TGF-β and melatonin were loaded into exosomes and used to treat skin problems (Figure 3). The loading methods used for genes often included electroporation and transfection with lipofectamine, while for small molecules and proteins, incubation methods were often used. Furthermore, to load genetic factors, the mother cell preloading method was frequently employed, and then exosomes containing the loaded cargo from modified cells were extracted and applied. While post-loading methods were used to load small molecule drugs, proteins, and growth factors. In addition, different methods of administering cargo-loaded exosomes were utilized in the studies on skin diseases, which complicates the standardization of exosome delivery approaches, making it difficult to achieve consistent therapeutic outcomes. Moreover, the reviewed studies alongside varying administration methods, lack consistent mention of the quantity of drug and exosomes used, and the extreme heterogeneity in experimental methodologies, including differences in dosage, tracking techniques, and exosome isolation methods, make it challenging to compare findings across studies. Nonetheless, the majority of studies indicate that delivering cargo via exosomes yielded superior results compared to administering the free drug alone for skin problems.

Although the delivery of varied bioactive molecules seems very promising for dermatological applications, there are multiple challenging issues in this field such as adequate efficiency, targeting specificity, and systemic circulation time. New research is trying to solve these problems, including specific targeting and therapeutic efficacy because the poor organ-targeting ability of exosomes led to decreased treatment efficacy and will limit their therapeutic applications. Recently, loading the exosomes by targeting magnetic nanoparticles captured the interest of the researchers. Studies showed that iron oxide nanoparticles-labeled exosomes enhance wound healing (Li X. et al., 2020; Wu et al., 2020). Indeed iron oxide increased the exosome accumulation at the wound site, and improved endothelial cell proliferation, migration, and tube formation while reducing scar formation and enhancing collagen deposition (Li X. et al., 2020). Therefore, exosomes modified with magnetic targeting nanoparticles may serve as a potential tool for therapeutic purposes. In addition, modification of exosomes with a variety of targeting ligands, stimuli-responsive, and immune evasion elements have been addressed to improve their drug delivery capability (Zhu et al., 2021; Fan et al., 2022). Recently, the method of surface functionalization with aptamer has been used to increase the targeting power of exosomes containing Dox in the treatment of melanoma mouse models (Chen H. et al., 2023). Furthermore, the use of PEG modification of functionalized exosomes in the targeted delivery of Dox in melanoma mouse models has been reported (Patras et al., 2022; Gao et al., 2024). Indeed, PEGylation of exosomes offers numerous benefits such as regulation of their pharmacokinetics and biodistribution, and improving circulation of blood half-time (Ferreira et al., 2022).

As a result, exosomes show significant potential as a successful method for delivering drugs to treat skin conditions. Although the practical application of exosomes as a drug delivery vehicle in skin diseases is still in its infancy, there is optimism regarding the potential clinical applications of exosomes in dermatology. Nevertheless, further research is required to elucidate the therapeutic effects of loaded exosomes on skin diseases.

## Glossary

**AD**: Atopic dermatitis
**ADSCs**: Adipose-derived stem cells
**AKT**: Protein kinase B
**ATV**: Atorvastatin
**ACEXO**: Anti-CD20 aptamer-modified exosomes
**BAX**: Bcl-2-associated X protein
**BMMSCs**: Bone marrow MSCs
**CARD10**: Caspase recruitment domain protein 10
**CA-exosomes**: Curcumin-albumin-exosomes
**CD31**: Cluster of differentiation 31
**CSCs**: Cancer stem cells
**cRGD**: cyclic RGD peptide
**DNA**: Deoxyribonucleic acid
**DFU**: Diabetic foot ulcer
**dMNAs**: Dissolvable microneedle arrays
**Dox**: Adriamycin/Doxorubicin
**dAExosome**: Dox-loaded ASL exosomes
**DR5**: Death receptor5
**ECM**: Extracellular matrix
**GelMA**: Gelatin methacrylate
**hAMSCs**: Human amnion MSCs
**hUCMSCs**: Human umbilical cord MSCs
**HUVECs**: Human umbilical vein endothelial cells
**HIF-1α**: Hypoxia-inducible factor-1α
**HaCaT**: Human keratinocyte cell line
iNOS: Inducible nitric oxide synthase
**JAK**: *Janus kinase*
**LPS**: Lipopolysaccharide
**LATS2**: Large tumor suppressor factor 2
**IL**: Interleukin
**lncRNA H19**: Long noncoding RNA H19
**IL-13-LCL-SIM**: Simvastatin incorporated in IL-13-functionalized long-circulating liposomes
**MSCs**: Mesenchymal stem cells
**miRNA:**: MicroRNA
**MEF2A**: Myocyte enhancer factor 2A
**mRNA**: Messenger RNA
**mDC**: Mature dendritic cell
**mDexos**: mature dendritic cell exosomes
**NOX**: NADPH Oxidase
**NF-κB**: Nuclear factor kappa B
**Nrf2**: Nuclear factor erythroid 2–related factor 2
**PEG**: Polyethylene glycol
**PGZ**: Pioglitazone
**PI3K**: Phosphoinositide-3-kinase
**PEG-EXO-DOX**: Dox-loaded PEGylated exosomes
**PD-1 receptors**: Programmed cell death protein one receptors
**PTEN**: Phosphatase and tensin homolog
**ROS**: Reactive oxygen species
**SEC**: Size exclusion chromatography
**siRNA**: Small interfering RNA
**SMSCs**: Synovial MSCs
**STAT3**: Signal transducer and activator of transcription 3
**siBRAF**: BRAF siRNA
**STZ**: Streptozotocin
**TSLP**: Thymic stromal lymphopoietin
**TLR4**: Toll-like receptor 4
**TGF-β**: Transforming growth factor beta
**TNF-α**: Tumor necrosis factor alpha
**TFC**: Tofacitinib
**Th17**: T helper 17
**Tp**: Triptolide
**TRAIL**: TNF-related apoptosis-inducing ligand
**TGFβRI**: Transforming growth factor-β receptor I
**VEGFR2**: Vascular endothelial growth factor receptor 2
**VEGF**: Vascular endothelial growth factor
**VEGFA**: Vascular Endothelial Growth Factor A
**VH298:**: Von Hippel-Lindau, the E3 ligase.

## References

[B1] Abdel-Megeed R. M. Ghanem H. Z. Kadry M. O. (2024). Alleviation of doxorubicin adverse effects via loading into various drug-delivery systems: a comparative study. Ther. Deliv. 15, 413–426. 10.4155/tde-2023-0121 38639647 PMC11285276

[B2] Akuma P. Okagu O. D. Udenigwe C. C. (2019). Naturally occurring exosome vesicles as potential delivery vehicle for bioactive compounds. Front. Sustain. Food Syst. 3, 23. 10.3389/fsufs.2019.00023

[B3] Anand V. Gupta S. Koundal D. (2022). “Skin disease diagnosis: challenges and opportunities,” in Proceedings of second doctoral symposium on computational intelligence: DoSCI 2021 (Springer), 449–459.

[B4] Aqil F. Munagala R. Jeyabalan J. Agrawal A. K. Gupta R. (2017). Exosomes for the enhanced tissue bioavailability and efficacy of curcumin. AAPS J. 19, 1691–1702. 10.1208/s12248-017-0154-9 29047044

[B5] Arntz O. J. Pieters B. C. Oliveira M. C. Broeren M. G. Bennink M. B. De Vries M. (2015). Oral administration of bovine milk derived extracellular vesicles attenuates arthritis in two mouse models. Mol. Nutr. and food Res. 59, 1701–1712. 10.1002/mnfr.201500222 26047123

[B6] Baeza A. Ruiz-Molina D. Vallet-Regí M. (2017). Recent advances in porous nanoparticles for drug delivery in antitumoral applications: inorganic nanoparticles and nanoscale metal-organic frameworks. Expert Opin. drug Deliv. 14, 783–796. 10.1080/17425247.2016.1229298 27575454

[B7] Bellavia D. Raimondo S. Calabrese G. Forte S. Cristaldi M. Patinella A. (2017). Interleukin 3-receptor targeted exosomes inhibit *in vitro* and *in vivo* Chronic Myelogenous Leukemia cell growth. Theranostics 7, 1333–1345. 10.7150/thno.17092 28435469 PMC5399597

[B8] Chen H. Jiang Y. Li X. (2023a). Adriamycin‐loaded exosome with anti‐CD20 aptamers selectively suppresses human CD20+ melanoma stem cells. Skin Res. Technol. 29, e13259. 10.1111/srt.13259 36704890 PMC9838758

[B9] Chen M. Wu M. Yaling Z. Zeng M., (2023b). Effect of MicroRNA-146a modified adipose-derived stem cell exosomes on rat back wound healing. J. of Lower Extremity Wound. 22, 704–712. 10.1177/15347346211038092 34459668

[B10] Cozmin M. Lungu I. I. Gutu C. Stefanache A. Duceac L. D. Șoltuzu B. D. (2024). Turmeric: from spice to cure. A review of the anti-cancer, radioprotective and anti-inflammatory effects of turmeric sourced compounds. Front. Nutr. 11, 1399888. 10.3389/fnut.2024.1399888 38863589 PMC11165187

[B11] Das C. K. Jena B. C. Banerjee I. Das S. Parekh A. Bhutia S. K. (2018). Exosome as a novel shuttle for delivery of therapeutics across biological barriers. Mol. Pharm. 16, 24–40. 10.1021/acs.molpharmaceut.8b00901 30513203

[B12] Dehghani P. Varshosaz J. Mirian M. Minaiyan M. Kazemi M. Bodaghi M. (2024). Keratinocyte exosomes for topical delivery of tofacitinib in treatment of psoriasis: an *in vitro*/*in vivo* study in animal model of psoriasis. Pharm. Res. 41, 263–279. 10.1007/s11095-023-03648-0 38263341 PMC10879239

[B13] Duan L. Xu L. Xu X. Qin Z. Zhou X. Xiao Y. (2021). Exosome-mediated delivery of gene vectors for gene therapy, Nanoscale, 13 **,** 1387–1397. 10.1039/d0nr07622h 33350419

[B14] El-Okaily M. S. Mostafa A. A. Dulnik J. Denis P. Sajkiewicz P. Mahmoud A. A. (2023). Nanofibrous polycaprolactone membrane with bioactive glass and atorvastatin for wound healing: preparation and characterization. Pharmaceutics 15, 1990. 10.3390/pharmaceutics15071990 37514176 PMC10384954

[B15] Escrevente C. Keller S. Altevogt P. Costa J. (2011). Interaction and uptake of exosomes by ovarian cancer cells. BMC cancer 11, 108–110. 10.1186/1471-2407-11-108 21439085 PMC3072949

[B16] Fan Z. Jiang C. Wang Y. Wang K. Marsh J. Zhang D. (2022). Engineered extracellular vesicles as intelligent nanosystems for next-generation nanomedicine. Nanoscale horizons 7, 682–714. 10.1039/d2nh00070a 35662310

[B17] Feng K. Li X. Bai Y. Zhang D. Tian L. (2024). Mechanisms of cancer cell death induction by triptolide: a comprehensive overview. Heliyon 10, e24335. 10.1016/j.heliyon.2024.e24335 38293343 PMC10826740

[B18] Ferreira D. Moreira J. N. Rodrigues L. R. J. C. R. I. O. H. (2022). New advances in exosome-based targeted drug delivery systems, Crit. Rev. Oncol. Hematol., 172 **,** 103628, 10.1016/j.critrevonc.2022.103628 35189326

[B19] Frost J. Galdeano C. Soares P. Gadd M. S. Grzes K. M. Ellis L. (2016). Potent and selective chemical probe of hypoxic signalling downstream of HIF-α hydroxylation via VHL inhibition. Nat. Commun. 7, 13312. 10.1038/ncomms13312 27811928 PMC5097156

[B20] Gao J. Karp J. M. Langer R. Joshi N. (2023). The future of drug delivery. Chem. Mater. 35 (2), 359–363. 37799624 10.1021/acs.chemmater.2c03003 PMC10553157

[B21] Gao S. Chen T. Hao Y. Zhang F. Tang X. Wang D. (2020). Exosomal miR-135a derived from human amnion mesenchymal stem cells promotes cutaneous wound healing in rats and fibroblast migration by directly inhibiting LATS2 expression. Stem Cell Res. Ther. 11, 56–11. 10.1186/s13287-020-1570-9 32054526 PMC7020560

[B22] Gao Y. Joshi M. Zhao Z. Mitragotri S. (2024). PEGylated therapeutics in the clinic. Bioeng. and Transl. Med. 9, e10600. 10.1002/btm2.10600 38193121 PMC10771556

[B23] Ge L. Wang K. Lin H. Tao E. Xia W. Wang F. (2023). Engineered exosomes derived from miR-132-overexpresssing adipose stem cells promoted diabetic wound healing and skin reconstruction. Front. Bioeng. Biotechnol. 11, 1129538. 10.3389/fbioe.2023.1129538 36937759 PMC10014603

[B24] Gilligan K. E. Dwyer R. M. (2017). Engineering exosomes for cancer therapy. Int. J. Mol. Sci. 18, 1122. 10.3390/ijms18061122 28538671 PMC5485946

[B25] Gondaliya P. Sayyed A. A. Bhat P. Mali M. Arya N. Khairnar A. (2022). Mesenchymal stem cell-derived exosomes loaded with miR-155 inhibitor ameliorate diabetic wound healing. Mol. Pharm. 19, 1294–1308. 10.1021/acs.molpharmaceut.1c00669 35294195

[B26] Gu Y. Du Y. Jiang L. Tang X. Li A. Zhao Y. (2022). αvβ3 integrin-specific exosomes engineered with cyclopeptide for targeted delivery of triptolide against malignant melanoma. J. nanobiotechnology 20, 384. 10.1186/s12951-022-01597-1 35999612 PMC9400227

[B27] Gurung S. Perocheau D. Touramanidou L. Baruteau J. (2021). The exosome journey: from biogenesis to uptake and intracellular signalling. Cell Commun. Signal. 19, 47. 10.1186/s12964-021-00730-1 33892745 PMC8063428

[B28] Hou C. Wu Q. Xu L. Cui R. Ou R. Li D. (2022). Exploiting the potential of extracellular vesicles as delivery vehicles for the treatment of melanoma. Front. Bioeng. Biotechnol. 10, 1054324. 10.3389/fbioe.2022.1054324 36466338 PMC9714302

[B29] Hu Y. Tao R. Chen L. Xiong Y. Xue H. Hu L. (2021). Exosomes derived from pioglitazone-pretreated MSCs accelerate diabetic wound healing through enhancing angiogenesis. J. nanobiotechnology 19, 150. 10.1186/s12951-021-00894-5 34020670 PMC8139165

[B30] Huang X.-Y. Huang Z.-L. Huang J. Xu B. Huang X.-Y. Xu Y.-H. (2020). Exosomal circRNA-100338 promotes hepatocellular carcinoma metastasis via enhancing invasiveness and angiogenesis. J. Exp. and Clin. Cancer Res. 39, 20–16. 10.1186/s13046-020-1529-9 31973767 PMC6979009

[B31] Jiang L. Gu Y. Du Y. Tang X. Wu X. Liu J. (2021). Engineering exosomes endowed with targeted delivery of triptolide for malignant melanoma therapy. ACS Appl. Mater. and Interfaces 13, 42411–42428. 10.1021/acsami.1c10325 34464081

[B32] Jonoush Z. A. Mahdavi R. Farahani M. Zeinali F. Shayan E. Amari A. (2024). The implications of exosomes in psoriasis: disease: emerging as new diagnostic markers and therapeutic targets. Mol. Biol. Rep. 51, 465. 10.1007/s11033-024-09449-x 38551769

[B33] Joorabloo A. Liu T. (2023). Engineering exosome-based biomimetic nanovehicles for wound healing. J. Control. Release 356, 463–480. 10.1016/j.jconrel.2023.03.013 36907562

[B34] Kang C. Han P. Lee J. S. Lee D. Kim D. (2020). Anchor, spacer, and ligand-modified engineered exosomes for trackable targeted therapy. Bioconjugate Chem. 31, 2541–2552. 10.1021/acs.bioconjchem.0c00483 33115231

[B35] Kang M. Jordan V. Blenkiron C. Chamley L. W. (2021). Biodistribution of extracellular vesicles following administration into animals: a systematic review. J. Extracell. vesicles 10, e12085. 10.1002/jev2.12085 34194679 PMC8224174

[B36] Kar R. Dhar R. Mukherjee S. Nag S. Gorai S. Mukerjee N. (2023). Exosome-based smart drug delivery tool for cancer theranostics. ACS biomaterials Sci. and Eng. 9, 577–594. 10.1021/acsbiomaterials.2c01329 PMC9930096 36621949

[B37] Keshtkar S. Azarpira N. Ghahremani M. H. J. S. C. R. (2018). Mesenchymal stem cell-derived extracellular vesicles: novel frontiers in regenerative medicine. Stem Cell Res. Ther. 9, 63–69. 10.1186/s13287-018-0791-7 29523213 PMC5845209

[B38] Keshtkar S. Kaviani M. Soleimanian S. Azarpira N. Asvar Z. Pakbaz S. J. F. I. M. (2022a). Stem cell-derived exosome as potential therapeutics for microbial diseases. Front. Microbiol. 12, 786111. 10.3389/fmicb.2021.786111 35237239 PMC8882917

[B39] Keshtkar S. Soleimanian S. Kaviani M. Sarvestani F. S. Azarpira N. Asvar Z. (2022b). Immune cell-derived extracellular vesicles in the face of pathogenic infections. Front. Immunol. 13, 906078. 10.3389/fimmu.2022.906078 35844564 PMC9279736

[B40] Khafaji M. Zamani M. Golizadeh M. Bavi O. (2019). Inorganic nanomaterials for chemo/photothermal therapy: a promising horizon on effective cancer treatment. Biophys. Rev. 11, 335–352. 10.1007/s12551-019-00532-3 31102198 PMC6557961

[B41] Kim H. Jang H. Cho H. Choi J. Hwang K. Y. Choi Y. (2021a). Recent advances in exosome-based drug delivery for cancer therapy. Cancers 13, 4435. 10.3390/cancers13174435 34503245 PMC8430743

[B42] Kim H. Lee J. W. Han G. Kim K. Yang Y. Kim S. H. J. P. (2021b). Extracellular vesicles as potential theranostic platforms for skin diseases and aging. Pharmaceutics 13, 760. 10.3390/pharmaceutics13050760 34065468 PMC8161370

[B43] Kim H. Wang S. Y. Kwak G. Yang Y. Kwon I. C. Kim S. H. (2019). Exosome‐guided phenotypic switch of M1 to M2 macrophages for cutaneous wound healing. Adv. Sci. 6, 1900513. 10.1002/advs.201900513 PMC6794619 31637157

[B44] Kim M. S. Haney M. J. Zhao Y. Mahajan V. Deygen I. Klyachko N. L. (2016). Development of exosome-encapsulated paclitaxel to overcome MDR in cancer cells. Nanomedicine Nanotechnol. Biol. Med. 12, 655–664. 10.1016/j.nano.2015.10.012 PMC4809755 26586551

[B45] Kim Y.-K. J. C. M. J. (2020). RNA therapy: current status and future potential, 56, 87. 10.4068/cmj.2020.56.2.87 PMC7250668 32509554

[B46] Kimiz-Gebologlu I. Oncel S. S. (2022). Exosomes: large-scale production, isolation, drug loading efficiency, and biodistribution and uptake. J. Control. Release 347, 533–543. 10.1016/j.jconrel.2022.05.027 35597405

[B47] Kooijmans S. Fliervoet L. Van Der Meel R. Fens M. Heijnen H. En Henegouwen P. V. B. (2016). PEGylated and targeted extracellular vesicles display enhanced cell specificity and circulation time. J. Control. Release 224, 77–85. 10.1016/j.jconrel.2016.01.009 26773767

[B48] Kumar D. N. Chaudhuri A. Dehari D. Gamper A. M. Kumar D. Agrawal A. K. (2024). Enhanced therapeutic efficacy against melanoma through exosomal delivery of hesperidin. Mol. Pharm. 21, 3061–3076. 10.1021/acs.molpharmaceut.4c00490 38757678

[B49] Lee J. H. Song J. Kim I. G. You G. Kim H. Ahn J.-H. (2022). Exosome-mediated delivery of transforming growth factor-β receptor 1 kinase inhibitors and toll-like receptor 7/8 agonists for combination therapy of tumors. Acta Biomater. 141, 354–363. 10.1016/j.actbio.2022.01.005 35007784

[B50] Li B. Luan S. Chen J. Zhou Y. Wang T. Li Z. (2020a). The MSC-derived exosomal lncRNA H19 promotes wound healing in diabetic foot ulcers by upregulating PTEN via MicroRNA-152-3p, Mol. Ther. Nucleic Acids, 19 **,** 814–826. 10.1016/j.omtn.2019.11.034 31958697 PMC7005423

[B51] Li D. Zhang C. Gao Z. Xia N. Wu C. Liu C. (2023). Curcumin-loaded macrophage-derived exosomes effectively improve wound healing. Mol. Pharm. 20, 4453–4467. 10.1021/acs.molpharmaceut.3c00062 37525890

[B52] Li X. Liu L. Yang J. Yu Y. Chai J. Wang L. (2016). Exosome derived from human umbilical cord mesenchymal stem cell mediates MiR-181c attenuating burn-induced excessive inflammation, EBioMedicine, 8, 72–82. 10.1016/j.ebiom.2016.04.030 27428420 PMC4919539

[B53] Li X. Wang Y. Shi L. Li B. Li J. Wei Z. (2020b). Magnetic targeting enhances the cutaneous wound healing effects of human mesenchymal stem cell-derived iron oxide exosomes. J. nanobiotechnology 18, 113–114. 10.1186/s12951-020-00670-x 32799868 PMC7429707

[B54] Li X. Xie X. Lian W. Shi R. Han S. Zhang H. (2018). Exosomes from adipose-derived stem cells overexpressing Nrf2 accelerate cutaneous wound healing by promoting vascularization in a diabetic foot ulcer rat model, 50, 1–14. 10.1038/s12276-018-0058-5 PMC5938041 29651102

[B55] Liang G. Zhu Y. Ali D. J. Tian T. Xu H. Si K. (2020). Engineered exosomes for targeted co-delivery of miR-21 inhibitor and chemotherapeutics to reverse drug resistance in colon cancer. J. nanobiotechnology 18, 10–15. 10.1186/s12951-019-0563-2 31918721 PMC6950820

[B56] Liao K. Niu F. Dagur R. S. He M. Tian C. Hu G. (2020). Intranasal delivery of lincRNA-Cox2 siRNA loaded extracellular vesicles decreases lipopolysaccharide-induced microglial proliferation in mice. J. Neuroimmune Pharmacol. 15, 390–399. 10.1007/s11481-019-09864-z 31325121 PMC6980430

[B57] Lin J. Huang N. Li M. Zheng M. Wang Z. Zhang X. (2023). Dendritic cell-derived exosomes driven drug co-delivery biomimetic nanosystem for effective combination of malignant melanoma immunotherapy and gene therapy, 2087–2106. Development, and Therapy. 10.2147/DDDT.S414758 PMC10363389 37489176

[B58] Liu W. Yu M. Xie D. Wang L. Ye C. Zhu Q. (2020). Melatonin-stimulated MSC-derived exosomes improve diabetic wound healing through regulating macrophage M1 and M2 polarization by targeting the PTEN/AKT pathway. Stem cell Res. and Ther. 11, 1–15. 10.1186/s13287-020-01756-x 32600435 PMC7322868

[B59] Liu X. Lu Y. Xu Y. Hou S. Huang J. Wang B. (2019). Exosomal transfer of miR-501 confers doxorubicin resistance and tumorigenesis via targeting of BLID in gastric cancer. Cancer Lett. 459, 122–134. 10.1016/j.canlet.2019.05.035 31173853

[B60] Longevity O. J. O. M. Longevity C. (2023). Retracted: miR-124-3p delivered using exosomes attenuates the keratinocyte response to IL-17a stimulation in psoriasis, 9876751. 10.1155/2023/9876751 PMC10307268 37388522

[B61] Lv Q. Deng J. Chen Y. Wang Y. Liu B. Liu J. J. M. P. (2020). Engineered human adipose stem-cell-derived exosomes loaded with miR-21-5p to promote diabetic cutaneous wound healing. Mol. Pharm. 17, 1723–1733. 10.1021/acs.molpharmaceut.0c00177 32233440

[B62] Ma J. Zhang Z. Wang Y. Shen H. J. E. D. (2022). Investigation of miR-126-3p loaded on adipose stem cell-derived exosomes for wound healing of full-thickness skin defects, Exp. Dermatol., 31 **,** 362–374. 10.1111/exd.14480 34694648

[B63] Matsumoto A. Takahashi Y. Ariizumi R. Nishikawa M. Takakura Y. (2019). Development of DNA-anchored assembly of small extracellular vesicle for efficient antigen delivery to antigen presenting cells. Biomaterials 225, 119518. 10.1016/j.biomaterials.2019.119518 31586864

[B64] Mattos B. D. Rojas O. J. Magalhães W. L. (2017). Biogenic silica nanoparticles loaded with neem bark extract as green, slow-release biocide. J. Clean. Prod. 142, 4206–4213. 10.1016/j.jclepro.2016.11.183

[B65] Min Y. Caster J. M. Eblan M. J. Wang A. Z. (2015). Clinical translation of nanomedicine. Chem. Rev. 115, 11147–11190. 10.1021/acs.chemrev.5b00116 26088284 PMC4607605

[B66] Mittelbrunn M. Sánchez-Madrid F. (2012). Intercellular communication: diverse structures for exchange of genetic information. Nat. Rev. Mol. cell Biol. 13, 328–335. 10.1038/nrm3335 22510790 PMC3738855

[B67] Miyanishi M. Tada K. Koike M. Uchiyama Y. Kitamura T. Nagata S. (2007). Identification of Tim4 as a phosphatidylserine receptor. Nature 450, 435–439. 10.1038/nature06307 17960135

[B68] Morelli A. E. Larregina A. T. Shufesky W. J. Sullivan M. L. Stolz D. B. Papworth G. D. (2004). Endocytosis, intracellular sorting, and processing of exosomes by dendritic cells. Blood 104, 3257–3266. 10.1182/blood-2004-03-0824 15284116

[B69] Morishita M. Takahashi Y. Matsumoto A. Nishikawa M. Takakura Y. (2016). Exosome-based tumor antigens–adjuvant co-delivery utilizing genetically engineered tumor cell-derived exosomes with immunostimulatory CpG DNA. Biomaterials 111, 55–65. 10.1016/j.biomaterials.2016.09.031 27723556

[B70] Negrea G. Rauca V.-F. Meszaros M. S. Patras L. Luput L. Licarete E. (2022). Active tumor-targeting nano-formulations containing simvastatin and doxorubicin inhibit melanoma growth and angiogenesis. Front. Pharmacol. 13, 870347. 10.3389/fphar.2022.870347 35450036 PMC9016200

[B71] Nojehdehi S. Soudi S. Hesampour A. Rasouli S. Soleimani M. Hashemi S. M. (2018). Immunomodulatory effects of mesenchymal stem cell–derived exosomes on experimental type‐1 autoimmune diabetes. J. Cell. Biochem. 119, 9433–9443. 10.1002/jcb.27260 30074271

[B72] Obeid M. A. Al Qaraghuli M. M. Alsaadi M. Alzahrani A. R. Niwasabutra K. Ferro V. A. (2017). Delivering natural products and biotherapeutics to improve drug efficacy. Ther. Deliv. 8, 947–956. 10.4155/tde-2017-0060 29061102

[B73] Ortega A. Martinez-Arroyo O. Forner M. J. Cortes R. (2021). Exosomes as drug delivery systems: endogenous nanovehicles for treatment of systemic lupus erythematosus. Pharmaceutics 13, 3. 10.3390/pharmaceutics13010003 PMC7821934 33374908

[B74] Pascucci L. Coccè V. Bonomi A. Ami D. Ceccarelli P. Ciusani E. (2014). Paclitaxel is incorporated by mesenchymal stromal cells and released in exosomes that inhibit *in vitro* tumor growth: a new approach for drug delivery. J. Control. release 192, 262–270. 10.1016/j.jconrel.2014.07.042 25084218

[B75] Patras L. Ionescu A. E. Munteanu C. Hajdu R. Kosa A. Porfire A. (2022). Trojan horse treatment based on PEG-coated extracellular vesicles to deliver doxorubicin to melanoma *in vitro* and *in vivo* . Cancer Biol. and Ther. 23, 1–16. 10.1080/15384047.2021.2003656 PMC8812761 34964693

[B76] Prada I. Meldolesi J. (2016). Binding and fusion of extracellular vesicles to the plasma membrane of their cell targets. Int. J. Mol. Sci. 17, 1296. 10.3390/ijms17081296 27517914 PMC5000693

[B77] Qiu X. Liu J. Zheng C. Su Y. Bao L. Zhu B. (2020). Exosomes released from educated mesenchymal stem cells accelerate cutaneous wound healing via promoting angiogenesis. Cell Prolif. 53, e12830. 10.1111/cpr.12830 32608556 PMC7445410

[B78] Sarkar T. Sarkar S. Gangopadhyay D. N. J. I. J. O. D. (2020). Gene therapy and its application in dermatology, Indian J. dermatol., 65 **,** 341–350. 10.4103/ijd.IJD_323_20 33165431 PMC7640808

[B79] Sato Y. T. Umezaki K. Sawada S. Mukai S.-A. Sasaki Y. Harada N. (2016). Engineering hybrid exosomes by membrane fusion with liposomes. Sci. Rep. 6, 21933. 10.1038/srep21933 26911358 PMC4766490

[B80] Shamili F. H. Bayegi H. R. Salmasi Z. Sadri K. Mahmoudi M. Kalantari M. (2018). Exosomes derived from TRAIL-engineered mesenchymal stem cells with effective anti-tumor activity in a mouse melanoma model. Int. J. Pharm. 549, 218–229. 10.1016/j.ijpharm.2018.07.067 30075248

[B81] Shao S. Fang H. Li Q. Wang G. J. T. (2020). Extracellular vesicles in inflammatory skin disorders: from pathophysiology to treatment, Theranostics, 10 **,** 9937, 9955. 10.7150/thno.45488 32929326 PMC7481415

[B82] Sharma V. Mukhopadhyay C. D. (2024). Exosome as drug delivery system: current advancements. Extracell. Vesicle 3, 100032. 10.1016/j.vesic.2023.100032

[B83] Shi A. Li J. Qiu X. Sabbah M. Boroumand S. Huang T.C.-T. (2021). TGF-β loaded exosome enhances ischemic wound healing *in vitro* and *in vivo* . Theranostics 11, 6616–6631. 10.7150/thno.57701 33995680 PMC8120220

[B84] Shi C. Pei S. Ding Y. Tao C. Zhu Y. Peng Y. (2023). Exosomes with overexpressed miR 147a suppress angiogenesis and infammatory injury in an experimental model of atopic dermatitis. Sci. Rep. 13, 8904. 10.1038/s41598-023-34418-y 37264030 PMC10235063

[B85] Shi H. Xu X. Zhang B. Xu J. Pan Z. Gong A. (2017). 3, 3′-Diindolylmethane stimulates exosomal Wnt11 autocrine signaling in human umbilical cord mesenchymal stem cells to enhance wound healing. Theranostics 7, 1674–1688. 10.7150/thno.18082 28529644 PMC5436520

[B86] Shi L. Song D. Meng C. Cheng Y. Wang B. Yang Z. (2024). Opportunities and challenges of engineered exosomes for diabetic wound healing. Giant 18, 100251. 10.1016/j.giant.2024.100251

[B87] Sukhanova A. Bozrova S. Sokolov P. Berestovoy M. Karaulov A. Nabiev I. (2018). Dependence of nanoparticle toxicity on their physical and chemical properties. Nanoscale Res. Lett. 13, 44–21. 10.1186/s11671-018-2457-x 29417375 PMC5803171

[B88] Sun D. Zhuang X. Xiang X. Liu Y. Zhang S. Liu C. (2010). A novel nanoparticle drug delivery system: the anti-inflammatory activity of curcumin is enhanced when encapsulated in exosomes. Mol. Ther. 18, 1606–1614. 10.1038/mt.2010.105 20571541 PMC2956928

[B89] Sun X. Shan A. Wei Z. Xu B. (2018). Intravenous mesenchymal stem cell-derived exosomes ameliorate myocardial inflammation in the dilated cardiomyopathy. Biochem. biophysical Res. Commun. 503, 2611–2618. 10.1016/j.bbrc.2018.08.012 30126637

[B90] Svensson K. J. Christianson H. C. Wittrup A. Bourseau-Guilmain E. Lindqvist E. Svensson L. M. (2013). Exosome uptake depends on ERK1/2-heat shock protein 27 signaling and lipid Raft-mediated endocytosis negatively regulated by caveolin-1. J. Biol. Chem. 288, 17713–17724. 10.1074/jbc.M112.445403 23653359 PMC3682571

[B91] Talebi S. F. Kooshki A. Zarein M. Seify M. Dolatshahi B. Shoorei H. (2024). Protective effect of hesperidin on malathion-induced ovarian toxicity in mice: the role of miRNAs, inflammation, and apoptosis. Toxicol. Rep. 12, 469–476. 10.1016/j.toxrep.2024.04.003 40094084 PMC11907194

[B92] Tao S.-C. Guo S.-C. Li M. Ke Q.-F. Guo Y.-P. Zhang C.-Q. (2017). Chitosan wound dressings incorporating exosomes derived from MicroRNA-126-overexpressing Synovium mesenchymal stem cells provide sustained release of exosomes and heal full-thickness skin defects in a diabetic rat model, Stem Cells Transl. Med., 6 **,** 736–747. 10.5966/sctm.2016-0275 28297576 PMC5442792

[B93] Tian F. Chen Z. Xu T. (2019). Efficacy and safety of tofacitinib for the treatment of chronic plaque psoriasis: a systematic review and meta-analysis. J. Int. Med. Res. 47, 2342–2350. 10.1177/0300060519847414 31096817 PMC6567701

[B94] Tian T. Wang Y. Wang H. Zhu Z. Xiao Z. (2010). Visualizing of the cellular uptake and intracellular trafficking of exosomes by live‐cell microscopy. J. Cell. Biochem. 111, 488–496. 10.1002/jcb.22733 20533300

[B95] Wang J. Chen D. Ho E. A. (2021). Challenges in the development and establishment of exosome-based drug delivery systems. J. Control. Release 329, 894–906. 10.1016/j.jconrel.2020.10.020 33058934

[B96] Wang L. Abhange K. K. Wen Y. Chen Y. Xue F. Wang G. (2019). Preparation of engineered extracellular vesicles derived from human umbilical cord mesenchymal stem cells with ultrasonication for skin rejuvenation. ACS omega 4, 22638–22645. 10.1021/acsomega.9b03561 31909348 PMC6941387

[B97] Wang Y. Cao Z. Wei Q. Ma K. Hu W. Huang Q. (2022). VH298-loaded extracellular vesicles released from gelatin methacryloyl hydrogel facilitate diabetic wound healing by HIF-1α-mediated enhancement of angiogenesis. Acta Biomater. 147, 342–355. 10.1016/j.actbio.2022.05.018 35580827

[B98] Watkins R. Wu L. Zhang C. Davis R. M. Xu B. (2015). Natural product-based nanomedicine: recent advances and issues. Int. J. nanomedicine 10, 6055–6074. 10.2147/IJN.S92162 26451111 PMC4592057

[B99] Wu D. Kang L. Tian J. Wu Y. Liu J. Li Z. (2020). Exosomes derived from bone mesenchymal stem cells with the stimulation of Fe3O4 nanoparticles and static magnetic field enhance wound healing through upregulated miR-21-5p. Int. J. nanomedicine 15, 7979–7993. 10.2147/IJN.S275650 33116513 PMC7585514

[B100] Xi X.-M. Xia S.-J. Lu R. (2021). Drug loading techniques for exosome-based drug delivery systems. Die Pharmazie-An Int. J. Pharm. Sci. 76, 61–67. 10.1691/ph.2021.0128 33714281

[B101] Xu M. Yang Q. Sun X. Wang Y. (2020). Recent advancements in the loading and modification of therapeutic exosomes. Front. Bioeng. Biotechnol. 8, 586130. 10.3389/fbioe.2020.586130 33262977 PMC7686035

[B102] Yadav K. Sahu K. K. Yadav R. Raza W. Minz S. Singh M. R. (2024). A complex molecular landscape to drug delivery concept for achieving precise therapy in psoriasis. Med. Drug Discov. 22, 100183. 10.1016/j.medidd.2024.100183

[B103] Yerneni S. S. Yalcintas E. P. Smith J. D. Averick S. Campbell P. G. Ozdoganlar O. B. (2022). Skin-targeted delivery of extracellular vesicle-encapsulated curcumin using dissolvable microneedle arrays. Acta Biomater. 149, 198–212. 10.1016/j.actbio.2022.06.046 35809788

[B104] Yu H. Feng H. Zeng H. Wu Y. Zhang Q. Yu J. (2024). Exosomes: the emerging mechanisms and potential clinical applications in dermatology, Int. J. Biol. Sci., 20 **,** 1778, 1795. 10.7150/ijbs.92897 38481799 PMC10929203

[B105] Yu M. Liu W. Li J. Lu J. Lu H. Jia W. (2020). Exosomes derived from atorvastatin-pretreated MSC accelerate diabetic wound repair by enhancing angiogenesis via AKT/eNOS pathway. Stem cell Res. and Ther. 11, 1–17. 10.1186/s13287-020-01824-2 32787917 PMC7425015

[B106] Yuan R. Dai X. Li Y. Li C. Liu L. J. M. M. R. (2021). Exosomes from miR-29a-modified adipose-derived mesenchymal stem cells reduce excessive scar formation by inhibiting TGF-β2/Smad3 signaling, 24, 1–12. 10.3892/mmr.2021.12398 PMC8436211 34476508

[B107] Zeng H. Guo S. Ren X. Wu Z. Liu S. Yao X. J. C. (2023). Current strategies for exosome cargo loading and targeting delivery, Cells, 12 **,** 1416, 10.3390/cells12101416 37408250 PMC10216928

[B108] Zhang H. Wang Y. Bai M. Wang J. Zhu K. Liu R. (2018a). Exosomes serve as nanoparticles to suppress tumor growth and angiogenesis in gastric cancer by delivering hepatocyte growth factor si RNA. Cancer Sci. 109, 629–641. 10.1111/cas.13488 29285843 PMC5834801

[B109] Zhang X. Wang C. Wang J. Hu Q. Langworthy B. Ye Y. (2018b). PD‐1 blockade cellular vesicles for cancer immunotherapy. Adv. Mater. 30, 1707112. 10.1002/adma.201707112 29656492

[B110] Zhao H. Li Z. Wang Y. Zhou K. Li H. Bi S. (2023). Bioengineered MSC-derived exosomes in skin wound repair and regeneration. Front. Cell Dev. Biol. 11, 1029671. 10.3389/fcell.2023.1029671 36923255 PMC10009159

[B111] Zhou M. Weber S. R. Zhao Y. Chen H. Sundstrom J. M. (2020). Methods for exosome isolation and characterization. Exosomes, 23–38. 10.1016/b978-0-12-816053-4.00002-x

[B112] Zhu M. Li S. Li S. Wang H. Xu J. Wang Y. (2021). Strategies for engineering exosomes and their applications in drug delivery. J. Biomed. Nanotechnol. 17, 2271–2297. 10.1166/jbn.2021.3196 34974854

[B113] Zhuang X. Xiang X. Grizzle W. Sun D. Zhang S. Axtell R. C. (2011). Treatment of brain inflammatory diseases by delivering exosome encapsulated anti-inflammatory drugs from the nasal region to the brain. Mol. Ther. 19, 1769–1779. 10.1038/mt.2011.164 21915101 PMC3188748

